# Treasure of the Past VI: Standard Potential of the Silver-Silver-Chloride Electrode from 0° to 95° C and the Thermodynamic Properties of Dilute Hydrochloric Acid Solutions

**DOI:** 10.6028/jres.106.021

**Published:** 2001-04-01

**Authors:** Roger G. Bates, Vincent E. Bower

## Abstract

From electromotive-force measurements of the cell without liquid junction:

$$\text{Pt};\;{\text{H}}_{2},\;\text{HCl}\;(m),\;\text{AgCl};\;\text{Ag}$$

through the range 0° to 95° C, calculations have been made of (1) the standard potential of the silver–silver-chloride electrode, (2) the activity coefficient of hydrochloric acid in aqueous solutions from *m* (molality) =0 to *m*=0.1 and from 0° to 90° C, (3) the relative partial molal heat content of hydrochloric acid, and (4) the relative partial molal heat capacity of hydrochloric acid.

The extrapolations were made by the method of least squares with the aid of punch-card techniques. Data from at least 24 cells were analyzed at each temperature, and 81 cells were studied at 25° C. The value of the standard potential was found to be 0.22234 absolute volt at 25° C, and the standard deviation was 0.02 millivolt at 0° C, 0.01 millivolt at 25° C, and 0.09 millivolt at 95° C. The results from 0° to 60° C are compared with earlier determinations of the standard potential and other quantities derived from the electromotive force.

## 1. Introduction

The silver–silver-chloride electrode is employed extensively in the determination of ionization constants and other thermodynamic data by the electromotive-force method [1].^1^ It is therefore important that the standard potential of this electrode be known as accurately as possible over a wide range of temperature.

Electromotive-force measurements of cell A

$$\text{Pt};H_{2}(g,1\;\text{atm}),\text{HCl}\;(m),\text{AgCl};\text{Ag},$$ (A)

at values of *m* sufficiently low to be useful in determining the standard potential by extrapolation to zero molality have been made by a number of investigators [2 to 16].^2^ The measurements of Güntelberg were made at 20° C, and all of the other investigations, except that of Harned and Ehlers which covered the range 0° to 60° C, were confined to 25° C. Recently, Harned and Paxton [17] have calculated the standard potential for the range 0° to 50° C from the electromotive force of cells of type A containing aqueous mixtures of hydrochloric acid and strontium chloride. In connection with the establishment of pH standards, the standard potential was needed in the range 60° to 95° C. In view of the extensive use of this electrode in electrochemical studies, it was deemed desirable to redetermine the standard potential at lower temperatures as well.

The measurements reported here were made at 17 temperatures from 0° to 95° C and were limited to molalities between 0.001 and 0.12. The number of cells studied ranged from 24 at 45° C and 55° C to 80 at 60° C and 81 at 25° C. The equations used for extrapolation were obtained by the method of least squares. Punchcard techniques aided in the calculation.

## 2. Experimental Procedures

Hydrochloric acid of reagent grade was distilled in an all-glass still; the middle fraction (about two-thirds) of the distillate was collected and redistilled. The middle fraction of the distillate from the second distillation was diluted, as needed, with water to about 0.1 *m* and was standardized gravimetrically by weighing silver chloride. Test of the undiluted acid revealed no bromide [18]. One of the three 0.1-*m* stock solutions was standardized three times over a period of 8 months; the concentration appeared to have changed only 0.02 percent in that time.

The cell solutions were prepared as needed by diluting portions of the stock solutions with water that had a conductivity of about 0.8 × 10^−6^ ohm^−1^ cm^−1^ at room temperature. Dissolved air was removed from most of the solutions by bubbling nitrogen; the rest of the solutions were saturated with hydrogen or boiled under vacuum. When the latter procedure was used, the weight of the solution was determined after boiling so that the final concentration could be calculated accurately. The electrolytic hydrogen, obtained in cylinders, was purified by passage over a platinum catalyst at room temperature and then over copper at 500° C.

Each of the cells, described elsewhere [19], contained two hydrogen electrodes and two silver–silver-chloride electrodes. The latter were of the thermal-electrolytic type [2, 20]. The silver oxide from which they were prepared was washed 40 times with distilled water. The 1-*M* hydrochloric acid in which they were chloridized was a distilled sample free of bromide. The electrodes were prepared at least 24 hours before use. For the high-temperature series (60° to 95° C), the cells were provided with extra hydrogen saturators consisting of three chambers, as described by Bates and Pinching [21].

Two calibrated potentiometers were used. The standards of electromotive force were a pair of saturated Weston cells maintained at a temperature near 36° C in a thermostated box of the type described by Mueller and Stimson [22]. Three constant-temperature baths were employed; water baths were used from 0° to 60° C and an oil bath from.60° to 95° C. The temperature was regulated to the desired even temperature within the limits of ±0.02 deg C from 25° to 80° C and ±0.03 deg C from 0° to 20° C and above 80° C. Temperature measurements were made with a platinum resistance thermometer. The difference of temperature between the oil bath and the solution in a cell immersed in the bath was found to be less than 0.1 deg C at 90° C.

The cells from which the data for the range 0° to 60° C were obtained were measured initially at 25° C. The constant-temperature water thermostat was lowered to near 0° C overnight, and the measurements from 0° to 30° C were made on the second day, followed on the third day by the measurements from 30° to 60° C. A final check of 34 of the cells was made at 25° C. The average difference between initial and final values was 0.18 mv. The final value was almost always lower than the initial value, and there was some indication that a considerable time was required for equilibrium to be established after the rapid drop from the higher temperature. Seven of the cells were measured only in the range 25° to 60° C. The data for the high range, 60° to 95° C, were obtained from a separate group of cells immersed in an oil bath. The initial measurements of these cells were made at 25° C or at 60° C, and the other temperatures were studied in ascending order. A final check at 60° C was sometimes but not always made.

The electromotive-force values were corrected to a partial pressure of hydrogen of 1 atm. Inasmuch as the ionic strength did not exceed 0.113, the vapor pressure of each solution from 0° to 70° C was taken to be that of pure water [23]. The error introduced by this approximation appears to be less than 0.02 mv at 70° C for the most concentrated solution studied. At 80°, 90°, and 95° C, the pressure correction was made with sufficient accuracy by assuming that the relative vapor-pressure lowering due to the presence of hydrochloric acid is the same as at 25° C [24].

Hills and Ives [25] have identified an excess pressure effect due to the depth of the jet through which the hydrogen enters the solution. From their results, it is evident that the effective partial pressure of hydrogen at an electrode located just below the surface is greater than that in the gas phase by (0.4 *h*/13.6) mm, where *h* is the depth in millimeters of the hydrogen jet below the surface. In the cells used in this work, *h* was about 40 mm. The correction therefore amounts to 0.02 mv at 25° C, 0.03 mv at 60° C, 0.08 mv at 90° C, and 0.16 mv at 95° C. Nevertheless, the corrections were not applied to the electromotive-force data and standard potentials reported here, in order that these results could be used directly in other studies where the average jet depth is about the same (namely, 4 cm) as in this investigation. The thermodynamic constants for hydrochloric acid solutions are unaffected, as they depend upon the difference *E*–*E*° and its change with temperature.

## 3. Standard Potential of the Cell

From the equation for the electromotive force, *E*, of cell A one can write

$$E{}^{\circ}=E+\frac{4.60518RT}{F}(\log\;m+\log\;\gamma_{\pm }),$$ (1)

where *E*° is the standard potential of the cell, γ_±_ is the stoichiometric mean ionic molal activity co-efficient of hydrochloric acid, and the other symbols have their usual significance.. Harned and Owen [1, chap. 11] have shown that experimental activity coefficients of uni-univalent strong electrolytes up to 1 *m* can be expressed with high accuracy by an equation of the form

$$\log\;\gamma_{\pm }=\frac{-A\sqrt{c}}{1+Ba*\sqrt{c}}+Cc+(ext.)-\log(1+0.03604\;m),$$ (2)

where *c* is the molar concentration, *A* and *B* are constants of the Debye-Hückel theory, *C* is an adjustable parameter, and a* is the ion-size parameter, and (*ext*.) represents the total contribution of the extended terms in the Debye-Hückel theory.

When *m* does not exceed 0.1, $\sqrt{c}$ differs from $\sqrt{md{}^{\circ}}$, where *d*° is the density of pure water, by less than 1 part in 1,000. Substitution of *md*° for *c* in eq (2) and combination with eq (1) gives

$$E{{}^{\circ}}^{\prime \prime }\equiv E{}^{\circ}-\beta m=E+\frac{4.60518RT}{F}\left[\log\;m-\frac{A^{\prime }\sqrt{m}}{1+B^{\prime }a*\sqrt{m}}+(ext.)-\log(1+0.03604\;m)\right],$$ (3)

where *β* is a constant for a particular temperature and value of a*. The values of *A′* and *B′* from 0° to 100° C have been tabulated elsewhere [26], and *(ext.)* from 0° to 60° C for a*=4.3 is given by Harned and Ehlers [9]. The latter is only −0.00075 at 0° and −0.00094 at 60° for the highest concentration studied in this investigation; hence, its value for 70°, 80°, 90°, and 95° C was obtained by linear extrapolation. These values of (*ext*.) were used in the calculations at all the temperatures studied. The extended terms correction becomes 0 at *m*=0, but is a function of a*. The differences, *ext.* (4.3 A)–*ext*. (6.0 A), at 60° C (where the best fit was obtained with a*=6.0) were not quite linear with *m*. Nevertheless, the mean departure from a straight line was less than ±0.03 mv, or about one-third the probable error at this temperature. The values of 2.30259*RT/F* in absolute volts were computed from *R*=8.31439 j deg^−1^ mole^−1^ and *F*=96493.1 coulombs equivalent^−1^ [27], and the absolute temperature, *T*, was taken to be *t*° C+ 273.160.

**Figure 1:**
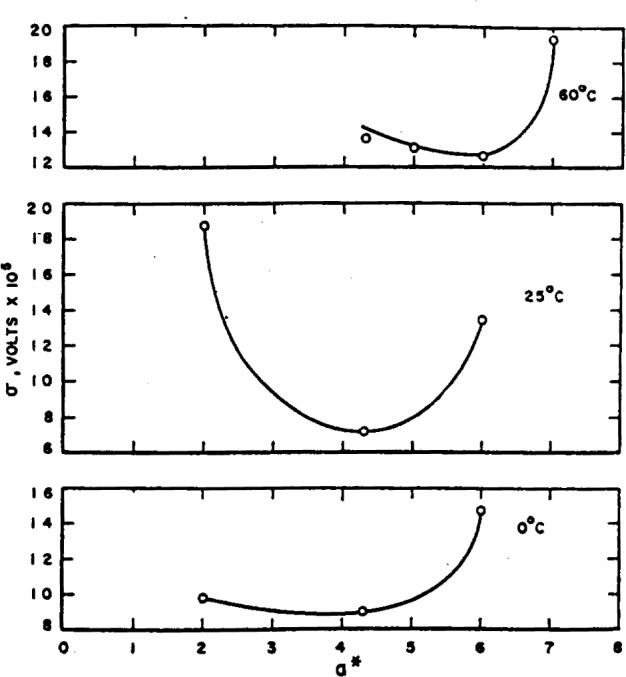
Standard deviation of E°″ from the least-square line as a function of a* at 0°, 25°, and 60° C.

The number of solutions studied was sufficiently large to justify the use of statistical procedures in analyzing the data. With the proper choice of a*, a plot of *E*°″, eq (3), should be a straight line with intercept *E*° and slope *−β*. The best value of a* is presumably tbe one that makes *E*°″ most nearly a linear function of *m*. To ascertain this best value, *E*°″ was calculated for three values of a* at 0°, 25°, and 60° C and fitted to a linear equation by the method of least squares. The standard deviation, *σ*, of an experimental point from the least-square line is plotted as a function of a* in figure 1. The curves are believed to justify the selection of 4.3 A for a* at 0° and 25° C and 6.0 A at 60° C. The values of a* for temperatures between 25° and 60° and from 70° to 95° C were determined by inspection of the plots of *E*°″ as a function of *m* for two or more values of a*.

If an incorrect value of the ion-size parameter is used, the plots of *E*°″ with respect to *m* become curved, and the intercept of the straight line established by least squares is no longer the true value of *E*°. The influence of a change in a* is demonstrated by the data for 25° C:

**Table:** 

a*	*E°*	*σ*
		
A	*v*	*mv*
2.0	0.22222	0.19
4.3	.22234	.07
6.0	.22246	.13

Table 1 contains a summary of the least-square calculations at the 17 temperatures. The standard potential of cell A is given in the fifth column. The standard deviation, *σ_i_*, in millivolts, of the intercept is given in the sixth column. The value of *E*° from 0° to 90° C is given by the equation

$$E{}^{\circ}=0.23659-(4.8564\times 10^{-4})t-(3.4205\times 10^{-6})t^{2}+(5.869\times 10^{-9})t^{3},$$ (4)

where *t* is in degrees Celsius. The standard potential of the silver–silver-chloride electrode is either equal to *E*° (cell A) or *−E*°, depending on which of the two common conventions for single electrode potentials is adopted.

The “observed” values of *E*° are compared in table 1 with those calculated by eq (4). The last column gives Δ, the difference in millivolts, between the calculated and observed value at each temperature. The average value of Δ at the 16 temperatures is 0.04 mv.

**Figure 2:**
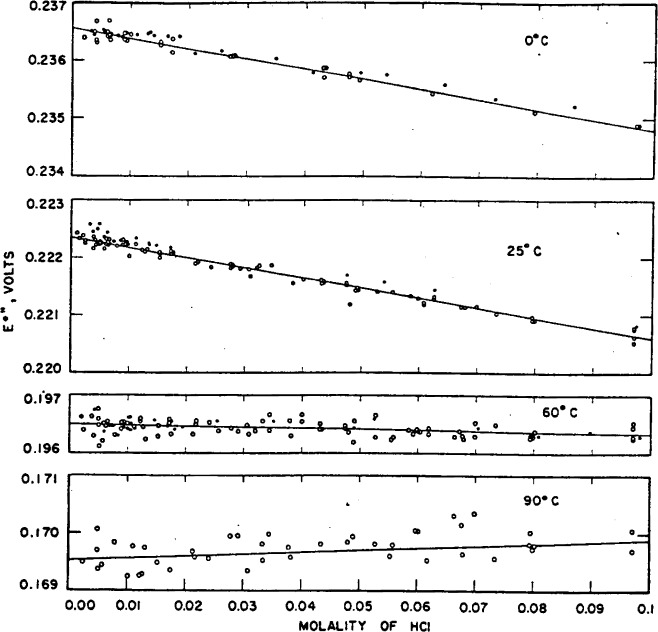
Plots of E°″ at 0°, 25°, 60°, and 90° C as a function of molality. Dots indicate the measurements of Harned and Ehlers.

Figure 2 is a plot of *E*°″ at 0°, 25°, 60°, and 90° C (open circles) as a function of molality. The closed circles were computed from the data of Harned and Ehlers [9] by the method described above. They lead to values of 0.23660 abs v for *E*° at 0°, 0.22252 v at 25°, and 0.19650 v at 60°.

## 4. Activity Coefficient of Hydrochloric Acid

The electromotive forces given in table 2 were computed from the smoothed values of *E*°″ at round values of the molality. This *E*°″ was computed, in turn, from the intercepts and slopes of the least-square lines listed in table 1. The mean activity coefficients calculated by eq (1) from these smoothed values of *E* and the values of *E*° given in table 1 are summarized in table 3.

Neither the electromotive force nor the activity coefficient was smoothed with respect to temperature. Hence, for a calculation of the thermodynamic quantities derived from the temperature coefficients of electromotive force, the values of −log *γ_±_* at 25° C and at intervals of 10 deg C from 0° to 90° were fitted by the method of least squares to a power series in *t*, the temperature on the Celsius scale:

$$-\log\;\gamma_{\pm }=A+Bt+Ct^{2}.$$ (5)

The values of log *γ_±_* were given equal weight at each temperature. The constants of this equation for eight values of the molality are listed in table 4. The last column gives the mean difference between the calculated and observed log *γ_±_* at the 11 temperatures, expressed as percentage of −log *γ_±_* at 25° C. When the values of log *γ_±_* were weighted according to the reciprocal of the probable error of *E*°″ at the appropriate temperature, the fit to eq (5) was not as complete as when equal weight was given to each value. The relative partial molal heat content computed from the two sets of constants differed on the average by 15 j mole^−1^ at 0° C, 7 j mole^−1^ at 25° C, and 42 j mole^−1^ at 90° C. The relative partial molal heat capacity was changed about 0.4 j deg^−1^ mole^−1^ at 0° C, 0.5 j deg^−1^ mole^−1^ at 25° C, and 1.2 j deg^−1^ mole^−1^ at 90° C.

## 5. Relative Partial Molal Heat Content and Heat Capacity

The temperature variation of log *γ_±_* can be used to calculate the partial molal beat content, ${\bar{L}}_{2}$, and partial molal heat capacity, ${\bar{J}}_{2}$, of hydrochloric acid relative to its value in the infinitely dilute solution. The former is given by

$$\frac{\partial (-\log\;\gamma_{\pm })}{\partial T}=\frac{{\bar{L}}_{2}}{4.6052T^{2}},$$ (6)

where *T* is the temperature on the Kelvin scale. Inasmuch as *∂T=∂t*, we obtain, by combination of eq (5) and (6),

$${\bar{L}}_{2}=4.6052RT^{2}(B+2Ct)$$ (7)

and

$${\bar{J}}_{2}=\frac{\partial {\bar{L}}_{2}}{\partial T}=9.2104RT^{2}C+9.2104RT(B+2Ct).$$ (8)

The values of ${\bar{L}}_{2}$ and ${\bar{J}}_{2}$, in absolute joules, calculated from these two equations are listed in tables 5 and 6.

**Figure 3:**
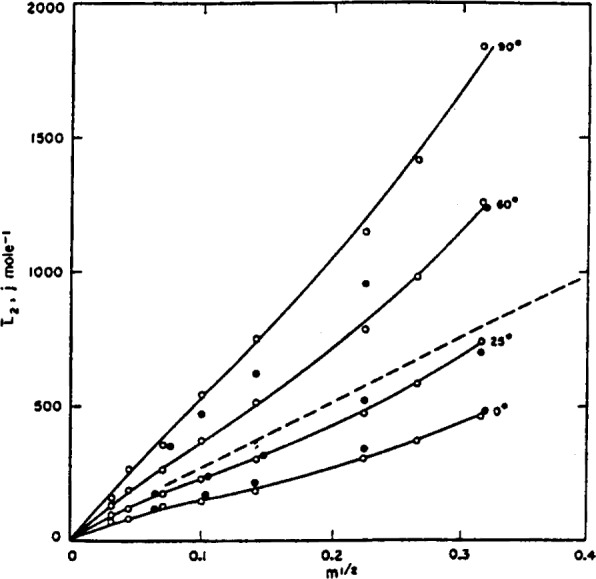
Relative partial molal heat content, ${\bar{L}}_{2}$, of hydrochloric acid at 0°, 25°, 60°, and 90° C as a function of the square root of the molality. Closed circles indicate the results of Harned and Ehlers. Dashed line represents Sturtevant’s calorimetric results at 25° C.

**Figure 4:**
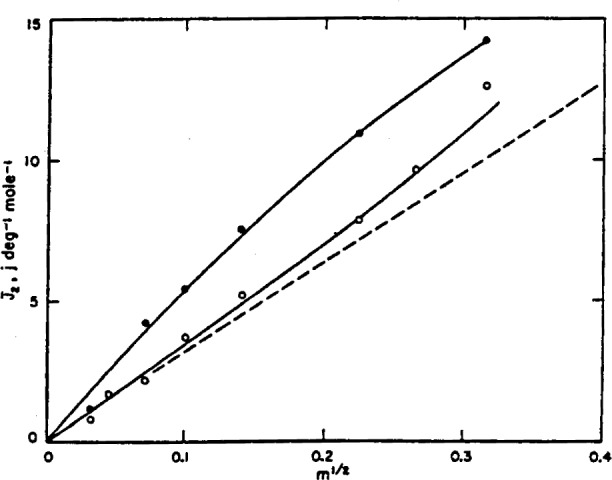
Relative partial molal heat capacity, ${\bar{J}}_{2}$, of hydrochloric acid at 25° C as a function of the square root of the molality. Closed circles indicate the values of Harned and Ehlers, and the dashed line is an extension of the calorimetric data obtained by Gucker and Schminke above 0.1 m.

The relative partial molal heat content at 0°, 25°, 60°, and 90° C is plotted as a function of *m*^1/2^ in figure 3. The dots represent the results obtained by Harned and Ehlers [9, 1] at 0°, 25°, and 60° C. The dashed line locates Sturtevant’s calorimetric values at 25° C [28]. The agreement with the earlier determinations can be regarded as very satisfactory at 0° and 25° C and acceptable at 60° C. The relative partial molal heat capacity, ${\bar{J}}_{2}$, at 25° C is plotted in figure 4. The dots again indicate the values obtained from the measurement of Harned and Ehlers.^3^ The dashed line is an extension to *m*^1/2^=0 of the straight line representing the values of ${\bar{J}}_{2}$ obtained calorimetrically by Gucker and Schminke [29]^4^ at molalities from 0.1 to 2.25.

## 6. Discussion

The values of *E*° for the temperature range 0° to 60° C are compared in table 7 with those obtained from the measurements of Harned and coworkers [9, 17]. The standard potentials of Harned and Paxton, given in the fifth column, are in better agreement with the present work than are those of Harned and Owen (second column). Although their values are based on only six points below an ionic strength of 0.1, Harned and Paxton point out that a straight line could be drawn to within 0.03 mv of these six points at nearly every temperature.

Harned and Wright’s recalculation [10] of Harned and Ehlers’ data, based on improved values of the natural constants, lowered the figures in the second column of table 7 by an average of about 0.14 mv (0° to 40° C), whereas Swinehart’s recent recalculation [14] with the aid of newer values of *R*, *T*, and *F*, raised them by 0.09 mv on the average. The extrapolation method of Harned and Elders, which expressed the activity coefficient in eq (1) by the Debye-Hückel limiting law, was used to obtain all of the potentials except those given in the last column. In the present investigation it was found that such an extrapolation procedure, applied to data at molalities up to 0.1, yields a curved line, concave upward, and of appreciable slope at low concentrations.

Evidently the consistency of the different sets of data can only be judged if both sets are treated in the same manner. As may be seen in figure 2, the electromotive-force data and standard potentials reported here are in acceptable agreement with those of Harned and Ehlers at 0° and 60° C, but appear to be about 0.18 mv lower at 25° C. A difference of this magnitude at 25° C, where the results are statistically the most precise, is difficult to explain, particularly because the silver–silver-chloride electrodes and the hydrochloric acid were prepared by similar procedures in the two investigations. A critical examination of the electromotive-force data obtained by other workers is therefore of particular interest.

**Figure 5:**
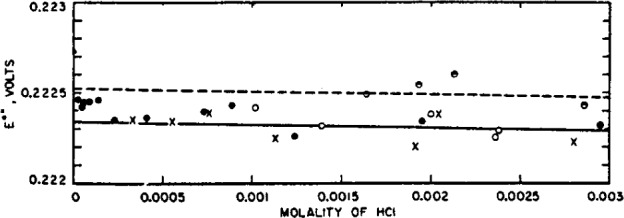
Plot of E°″ at low concentrations as a function of molality at 25° C. Circles, data of this investigation; closed circles, data of Anderson and Young; crosses, data of Carmody; half-shaded circles, data of Roberts.

This comparison was made first at low concentrations, where the mode of extrapolation has the smallest influence on the result. All of the available emf data were accordingly converted to absolute volts by multiplying by 1.00033 [30]. Values of *E*°″ were then computed by eq (3) with a*=4.3. The results of this recalculation at molalities below 0.003 are shown in figure 5. The open circles are the data of this investigation, and the least square line is shown. The dashed line is the extension of the straight line through the points of Harned and Ehlers, all of which were at molalities above 0.003.

The most numerous data in this region of low concentrations are those of Anderson and Young [15], indicated by closed circles in the figure. The value of *E*° obtained from these measurements appears to be about 0.22242 abs v. The crosses were calculated from the measurements of Carmody |8], and the half-shaded circles mark the lowest points of Roberts [7]. The other data for cell A in this low-range display larger deviations and are not plotted. The four measurements of Linhart [3] in the range of the figure, all below 0.001 *m*, vary from 0.2225 to 0.2228. The five points of Maronny and Valensi [16] below 0.0025 *m* lie 0.1 to 0.4 mv below the solid line. The average value of *E*°″ computed from Nonhebel’s six measurements [5] between *m*=0.0008 and *m*=0.003 is 0.22243 ±0.00005 abs v. Below *m*=0.0008, however, *E*°″ rises rapidly, exceeding 0.223 v at the lowest molalities studied.

A comparison limited to low concentrations suffers from the fact that the experimental data are usually less accurate below 0.01 *m* than above. Hence, the electromotive-force data of Güntelberg [6] at 20° and of Roberts, Carmody, Harned and Ehlers, and Anderson and Young at 25° C for molalities up to 0.1 *m* were smoothed to round molalities, where necessary, on a plot of *E*°″ as a function of *m* and are compared in table 8. It is seen that the values of Güntelberg agree reasonably well with those reported here and are somewhat lower than those of Harned and Ehlers. The latter are also higher than the others at 25° C, whereas those of Carmody and of Anderson and Young agree well with the present work. The emf data obtained by Roberts between 0.01 *m* and 0.1 *m* appear to fall between those of this investigation and the data of Harned and Ehlers. With the exception of one low value, obviously erroneous, the five points of Noyes and Ellis [2] below *m*=0.1 agree with the results reported here, as do the four of Scatchard [4] between 0.0104 and 0.1 *m* and the two of Linhart [3] above 0.01 *m*. Scatchard’s three points near 0.01 m, however, lie nearly 0.2 mv below the line through his other points. It may be concluded that the work of Güntelberg, Carmody, and of Anderson and Young is consistent with the present study, whereas the measurements of Nonhebel and Roberts, and those of Linhart below 0.01 *m*, tend to support the higher value of Harned and Ehlers at 25° C.

A rather large upward trend in *E*°″ at the lowest concentrations is observed in the data of Linhart as well as of Nonhebel. A departure from the theoretical slope is not to be expected in this region and was not found by Carmody or by Anderson and Young. It is possible that traces of oxygen, known to shift the potential of the silver–silver-chloride electrode toward more positive values in acid solutions, may explain this elevation of electromotive force at low molalities. The chloride-ion concentration in the vicinity of the silver–silver-chloride electrode is lowered by the following reaction [6]:

$$2\;\text{Ag}+2\;\text{HCl}+O=2\;\text{AgCl}+H_{2}O\text{.}$$ (9)

The resulting change of emf may be appreciable in dilute solutions, for *dE/dn*_Cl_, where *n*_Cl_ is a number of equivalents of chloride ion, is much larger than in solutions of moderate or high concentration.

Nevertheless, dissolved air cannot explain the difference between the results of the present investigation and those of Harned and Ehlers because an air-free technique was used in both investigations. The potentials of silver–silver-chloride electrodes are known to be altered likewise by traces of bromide [18, 31] and by aging during the first 30 hours after preparation [32]. A lowering of the electromotive force of the cell by 0.18 mv would require about 0.02 mole percent of bromide impurity in the hydrochloric acid used in this study; this quantity could hardly have gone undetected in the test that was performed. The effect due to aging causes the emf of the cell containing a freshly prepared silver–silver-chloride electrode to be too high. The agreement among measurements at 25° C made at different points in the temperature series would seem to rule out a pronounced effect due to aging. No simple reasonable explanation for the differences between emf values at 25° C reported here and those of Harned and Ehlers has been found.

The activity coefficients and other thermodynamic properties of hydrochloric acid are dependent not upon the value of *E*° but on the difference *E–E*°. Inasmuch as the extrapolation lines are nearly parallel (see fig. 2),^5^ the activity coefficients at 25° C reported here agree very well with those computed from Harned and Ehlers’s measurements with a standard potential of 0.22252 abs v (the value obtained from the emf data of Harned and Ehlers by the extrapolation procedure used in the present work). The activity coefficients from these two sources are compared in table 9 with those obtained by Hills and Ives [13] in a careful study of the hydrogen-calomel cell without liquid junction and with those computed by Shedlovsky [33]^6^ from transference numbers and the electromotive force of cells with transference. The agreement with the determination of Hills and Ives is very satisfactory, and the only notable difference from the values of Shedlovsky appears to be at *m*=0.1, where the departure corresponds to 0.19 mv in the electromotive force.

## Figures and Tables

**Table 1:** The Standard potential of the cell: H_2_; HCl (m), AgCl; Ag from 0° to 95° C

*t*	Number of cells	a*	*β*	*E*°	*σ_i_*	*E*° (eq 4)	Δ

*°C*				*abs v*	*mv*	*abs v*	*mv*
0	31	4.3	1.74×10^2^	0.23655	0.02	0.23659	+0.04
5	31	4.3	1.80	.23413	.02	.23408	−.05
10	32	4.3	1.79	.23142	.01	.23140	−.02
15	32	4.3	1.79	.22857	.01	.22856	−.01
20	32	4.3	1.82	.22557	.02	.22557	.00
25	81	4.3	1.75	.22234	.01	.22240	+.06
30	44	4.3	1.75	.21904	.02	.21910	+.06
35	37	5.0	1.15	.21565	.02	.21566	+.01
40	37	5.0	1.23	.21208	.03	.21207	−.01
45	24	3.0	1.14	.20835	.03	.20834	−.01
50	32	5.0	1.09	.20449	.03	.20449	.00
55	24	5.0	1.12	.20056	.04	.20051	−.05
60	80	6.0	0.16	.19649	.03	.19641	−.08
70	43	6.0	−0.14	.18782	.04	.18785	+.03
80	49	6.0	−.37	.17873	.07	.17885	+.12
80	44	6.0	−.37	.16952	.06	.16946	−.06
95	37	6.0	−.32	.16511	.09	----------	----------

Summary of least-square calculations, and values of *E*° from 0° to 90° C calculated from eq (4).

**Table 2:** Smoothed values of the electromotive force of cell A in absolute volts from 0° to 90° C

*m*	*E*_0_	*E*_10_	*E*_20_	*E*_25_	*e*_30_	*E*_40_	*E*_50_	*e*_60_	*E*_70_	*E*_80_	*E*_90_

0.001	0.56330	0 57019	0.57631	0 57909	0.58178	0.58683	0.59125	0 59525	0 59860	0.6015	0.6043
.002	.53131	.53701	.54198	.54418	.54628	.55018	.55344	.55628	.55848	.5602	.5619
.005	.48931	.49351	.49695	.49840	.49977	.50211	.50388	.50517	.50589	.5062	.5063
.01	.45787	.46091	.46323	.46412	.46493	.46613	.46678	.46694	.46655	.4657	.4648
.02	.42669	.42853	.42985	.43019	.43044	.43049	.43006	.42909	.42764	.4258	.4238
.05	.38588	.38630	.38613	.38579	.38533	.38391	.38211	.37969	.37691	.3737	.3703
.07	.37094	.37089	.37016	.36957	.36885	.36691	.36461	.36174	.35848	.3548	.3509
.1	.35505	.35444	.35316	.35233	.35134	.34888	.34608	.34275	.33904	.3349	.3304

**Table 3:** Activity coefficient of hydrochloric acid from 0° to 90° C

*m*	0°	10°	20°	25°	30°	40°	50°	60°	70°	80°	90°

0.001	0.9670	0.9660	0.9654	0.9650	0.9648	0.9642	0.9635	0.9631	0.962	0.962	0.961
.002	.9540	.9533	.9524	.9520	.9518	.9507	.9499	.9493	.948	.947	.946
.005	.9313	.9299	.9289	.9283	.9274	.9268	.9252	.9249	.923	.921	.920
.01	.9081	.9069	.9054	.9045	.9034	.9026	.9006	.9000	.898	.895	.893
.02	.8805	.8786	.8766	.8753	.8741	.8735	.8707	.8700	.867	.863	.860
.05	.8381	.8357	.8331	.8308	.8291	.8283	.8239	.8227	.817	.813	.810
.07	.8223	.8196	.8163	.8137	.8119	.8107	.8058	.8033	.797	.792	.788
.1	.8067	.8038	.8000	.7967	.7946	.7927	.7867	.7828	.775	.769	.765

**Table 4:** Constants of the equation: −log γ_±_=A+Bt+Ct^2^ for the temperature range t=0° to t=90° C

*m*	*A*	*B*	*C*	Δ

				*Percent*
0.001	0.01470	0.273×10^4^	0.27×10^7^	0.39
.003	.02051	.288	1.30	.23
.005	.03106	.443	1.49	.34
.01	.04201	.510	3.13	.34
.02	.05556	.657	4.63	.49
.05	.07694	1.058	6.73	.42
.07	.08515	1.299	8.37	.38
.1	.09334	1.620	11.21	.44

Δ = mean difference between calculated and observed values, in percent of −log *γ_±_* at 25° C.

**Table 5:** Relative partial molal heat content, ${\bar{L}}_{2}$, of hydrochloric acid from 0° to 90° C

*m*	0°	10°	20°	25°	30°	40°	50°	60°	70°	80°	90°

0.001	78	85	93	98	102	111	120	130	140	151	163
.002	82	96	112	120	129	147	167	189	215	237	264
.005	127	145	166	176	187	211	237	264	294	325	359
.01	146	176	209	227	246	285	329	377	427	483	542
.02	188	230	277	303	329	386	448	516	588	668	752
.05	302	366	437	475	514	599	692	793	902	1,020	1,146
.07	371	450	538	585	634	739	854	979	1,114	1,260	1,417
.1	463	566	681	742	807	945	1,096	1,260	1,464	1,630	1,837

In abs j mole^−1^.

**Table 6:** Relative partial molal heat capacity, ${\bar{J}}_{2}$, of hydrochloric acid from 0° to 90° C

*m*	0°	25°	60°	90°

0.001	0.7	0.8	1.0	1.2
.002	1.3	1.7	2.2	2.8
.005	1.8	2.2	2.9	3.5
.01	2.9	3.7	4.9	6.1
.02	4.0	5.2	7.0	8.8
.05	6.1	7.8	10.5	13.1
.07	7.5	9.6	13.0	16.3
.1	9.8	12.6	17.1	21.4

In abs j deg^−1^ mole^−1^.

**Table 7:** Standard potential, E°, of cell A from 0° to 60° C, in absolute volts

*t*	Electromotive-force data of Harned and Ehlers [8]			Harned and Paxton [17]	This investigation
	Harned and Owen [1]	Harned and Wright [10] (recalculated)	Swinehart [14] (recalculated)		

*°C*					
0	0.23642	0.23635	0.23647	0.23652	0.23655
5	.23400	.23392	.23406	.23405	.23413
10	.23134	.23124	.23145	.23137	.23142
15	.22855	.22841	.22865	.22849	.22857
20	.22558	.22544	.22568	.22549	.22557
25	.22246	.22230	.22254	.22239	.22234
30	.21919	.21901	.21924	.21908	.21904
35	.21570	.21551	.21578	.21570	.21565
40	.21207	.21189	.21216	.21207	.21208
45	.20828	------------	.20841	.20833	.20835
50	.20444	------------	.20452	.20449	.20449
55	.20042	------------	.20050	----------	.20056
60	.19626	------------	.19635	----------	.19649

**Table 8:** Smoothed electromotive force of cell A at 20° and 25° C, in absolute volts

*m*	Güntelberg [6]	Roberts [7]	Carmody [8]	Harned and Ehlers [9]	Anderson and Young [15]	This investigation
Measurements at 20°						
0.01	0.46318	--------	--------	0.46334	--------	0.46323
.02	.42982	--------	--------	.42992	--------	.42985
.05	.38615	--------	--------	.38627	--------	.38613
.1	.35327	--------	--------	.35333	--------	.35316
Measurements at 25°						
0.001	--------	0.57921	0.57909	0.57931	0.57916	0.57909
.002	--------	.54429	.54418	.54441	.54422	.54418
.005	--------	.49852	.49842	.49859	--------	.49840
.01	--------	.46423	.46412	a.46433	--------	.46412
.02	--------	.43030	.43018	.43037	--------	.43019
.05	--------	.38590	.38581	.38600	--------	.38579
.1	--------	.35243	.35236	.35252	--------	.35233

a Harned and Paxton [17], 0.46437

**Table 9:** Activity coefficient of hydrochloric acid at 25° C

*m*	Harned and Ehlers [9]	Shedlovsky [33]	Hills and Ives [13]	This Investigation

0.001	0.9646	0 9653	0.9650	0.9650
.002	.9516	.9525	.9519	.9520
.005	.9285	.9287	.9280	.9283
.01	.9044	.9049	.9040	.9045
.02	.8755	.8757	.8747	.8753
.05	.8303	.8301	.8296	.8308
.07	--------	--------	.8129	.8137
.1	.7969	.7938	.7958	.7967

## Footnotes

1 Figures in brackets indicate the literature references at the end of this paper.

2 The calculation of the standard potential from the data of Harned and Ehlers has been examined by Harned and Wright [10], Prentiss and Scatchard [11], Hamer, Burton, and Acree [12], Hills and Ives [13], and Swinehart [14].

3 These are the means of the two sets of values given by Harned and Owen, computed from the experimental data in two different ways.

4 The data of Gucker and Schminke deviate sharply from the straight line below 0.1 m. This anomalous behavior has not been explained.

5 Prentiss and Scatchard [11] have noted that the slopes of the lines plotted from the data of Carmody, Roberts, and Harned and Ehlers are nearly the same.

6 A similar comparison has been made by King [34].
